# Long-Term High-Temperature Stability of Directionally Grown [Bi_2_Ba_2_O_4_]*_p_*[CoO_2_] Rods

**DOI:** 10.3390/ma10020146

**Published:** 2017-02-08

**Authors:** Juan C. Diez, María A. Madre, Miguel A. Torres, Shahed Rasekh, Andrés Sotelo

**Affiliations:** Instituto de Ciencia de Materiales de Aragón (CSIC-Universidad de Zaragoza), C/María de Luna 3, 50018 Zaragoza, Spain; amadre@unizar.es (M.A.M.); matorres@unizar.es (M.A.T.); shrasekh@unizar.es (S.R.); asotelo@unizar.es (A.S.)

**Keywords:** thermoelectricity, cobalt oxide, aging, flexural strength, power factor

## Abstract

[Bi_2_Ba_2_O_4_]*_p_*[CoO_2_] thermoelectric ceramics have been successfully grown from the melt using the laser floating zone method, followed by a thermal treatment at 700 °C under air between 0 and 1532 h. The microstructural, thermoelectric, and mechanical properties were evaluated as a function of the thermal treatment length. Microstructure has shown that as-grown samples are composed of thermoelectric grains, together with a relatively high amount of secondary phases. Thermal treatment decreased the number and amount of secondary phases, producing nearly single-phase samples after 384 h. Consequently, the thermoelectric properties evaluated through the power factor showed a slight increase with the thermal treatment length, mainly due to the decrease of electrical resistivity, while the Seebeck coefficient was nearly unchanged. On the other hand, flexural strength was practically constant after 24 h thermal treatment.

## 1. Introduction

Nowadays, energy transforming systems possess low efficiency, and more than 50% of their fuel consumption is released as wasted heat [1]. Thermoelectric (TE) materials can transform thermal to electrical energy without any moving parts, being able to harvest waste heat from a wide number of systems, increasing their efficiency, and consequently decreasing the CO_2_ emissions. Therefore, they can play a very important role in combating global warming. Furthermore, they can be used in green energy production, directly transforming solar energy into electricity at lower cost than photovoltaic technologies [2]. Usually, the performance of TE materials is quantified through the dimensionless figure of merit, *ZT*, defined as *TS*^2^/ρκ, where *T*, *S*, ρ, and κ are absolute temperature, Seebeck coefficient, electrical resistivity, and thermal conductivity, respectively [3]. From its definition, it is clear that in order to reach high *ZT* values, we need materials with a high *S* and low ρ and κ, able to work at high temperatures. On the other hand, the electrical part of this expression (*S*^2^/ρ) is called power factor, *PF*, and it has been shown that its optimization is essential in order to achieve high power density thermoelectric modules [4].

Today, some TE practical applications can be found in fields such as refrigeration, waste heat harvesting in automobile exhausts, or in radioisotope thermoelectric generators (RTG) [5,6]. Most of these TE devices are based on alloys and/or intermetallic thermoelectric materials such as Bi_2_Te_3_ or CoSb_3_, with high thermoelectric performances at relatively low temperatures [6,7]. On the other hand, these materials can be oxidized and/or degraded at high temperatures under air and/or release toxic or heavy elements. Consequently, their application range is limited to relatively low temperatures.

These temperature limitations were overtaken by the discovery of large thermoelectric properties in Na*_x_*CoO_2_ compound [8]. Since this time, great efforts have been made in the study of CoO-based materials, such as Ca-Co-O, Bi-Sr-Co-O, Bi-Ca-Co-O, or Bi-Ba-Co-O, with attractive TE properties [9,10,11,12,13,14,15,16]. The crystal structure of these oxides is composed of an alternate stacking of two different layers: a common conductive CdI_2_-type CoO_2_ layer with a two-dimensional triangular lattice, and a block one composed of insulating rock-salt-type (RS) layers. Both sublattices (RS block and CdI_2_-type CoO_2_ layer) possess common a- and c-axis lattice parameters and β angles, but different b-axis length, causing a misfit along the b-direction [17]. Consequently, these materials show a very important crystallographic anisotropy which influences their electrical properties. To overcome this problem, the alignment of grains along their conducting planes is usually used to obtain bulk textured materials with interesting TE properties. Various processes have been successfully applied to produce well-textured materials, such as spark plasma sintering [18], hot-pressing [19], laser floating zone (LFZ) [20], or the electrically-assisted laser floating zone [21].

In future practical applications, thermoelectric modules built using these Co-O based materials should support hard service conditions, mainly characterized by high temperatures in oxidative atmospheres (air). Accordingly, it is necessary to acquire a better knowledge about the thermal stability of these materials under air. Surprisingly, to the best of our knowledge there are very few studies dealing with these aspects in high working temperature thermoelectric materials [22,23,24].

In this work, the microstructural, thermoelectric, and mechanical evolution of textured [Bi_2_Ba_2_O_4_]*_p_*[CoO_2_] samples produced through the laser floating zone technique have been studied as a function of the thermal treatment length at 700 °C under air (between 0 and 1532 h).

## 2. Materials and Methods

The polycrystalline [Bi_2_Ba_2_O_4_]*_p_*[CoO_2_] ceramics used in this study have been prepared by the classical solid state route from commercial Bi_2_O_3_ (Panreac, Barcelona, Spain, 98 + %), BaCO_3_ (Panreac, 98 + %), and CoO (Panreac, 98%) powders. They were weighed in the appropriate proportions to account for *p* = 0.5 [13], and mixed using a planetary ball milling system at 300 rpm for 30 min in distilled water media. The resulting suspension was placed in a glass container and dried using infrared radiation. The dried powder was then thermally treated twice at 700 and 750 °C for 12 h under air with an intermediate manual milling in order to ensure the complete decomposition of the carbonates in the mixture. This thermal treatment is of the critical importance in LFZ technique in order to avoid the presence of carbonates in the precursors [20]. Otherwise, they would decompose in the molten zone, producing CO_2_ bubbles and leading to crystallization front destabilization. The resulting powders were then cold isostatically pressed into latex tubes at approximately 200 MPa for about 2 min to obtain green ceramic cylinders (φ ~3 mm and ~100 mm length). They were subsequently used as feed in an LFZ device equipped with a continuous power Nd:YAG laser (λ = 1064 nm) and optical focussing system described elsewhere [25]. All samples were processed under the same conditions: 30 mm/h downwards with a seed rotation of 3 rpm to help maintain cylindrical geometry. Moreover, in order to assure a better compositional homogeneity of the molten zone, an opposite rotation of 15 rpm was also performed on the feed. After the directional growth process, long (more than 12 cm) and geometrically homogeneous (~2 mm diameter) cylindrical rods were obtained. Finally, they were cut into suitably sized pieces for characterization (~15-mm-long specimens) and subsequently introduced to an electric furnace operating under air at 700 °C for different lengths of time (0, 12, 24, 48, 96, 192, 384, 768, and 1536 h).

Identification of the main phases was performed using powder X-ray diffraction (XRD) in a Rigaku D/max-B X-ray powder diffractometer (Tokyo, Japan) (CuKα radiation) in the range 2θ = 5°–60°. Microstructural evolution was observed employing a Zeiss Merlin field emission scanning electron microscope (FESEM) equipped with an energy dispersive spectrometer (EDS) (Carl Zeiss AG, Oberkochen, Germany), used to determine the elemental composition of the different phases. Longitudinal polished sections of all samples were observed to analyse the different phases, their orientation, and their distribution.

Mechanical characterization was performed by flexural strength, using the three-point bending test in an Instron 5565 machine (High Wycombe, UK) with a 10-mm loading span fixture and a punch displacement speed of 30 μm/min. The tests were carried out in a direction perpendicular to the cylindrical axis rods. Due to the ceramic nature of the samples, five specimens were tested for each condition to obtain more representative values. Electrical resistivity and Seebeck coefficient were simultaneously determined by the standard direct current (DC) four-probe technique in a LSR3 measurement system (Linseis GmbH, Selb, Germany) in the steady state mode, at temperatures ranging from near room temperature (~50 °C) to 650 °C. All thermoelectric properties were measured along the direction of the cylindrical axis rods.

## 3. Results and Discussion

**Figure 1:**
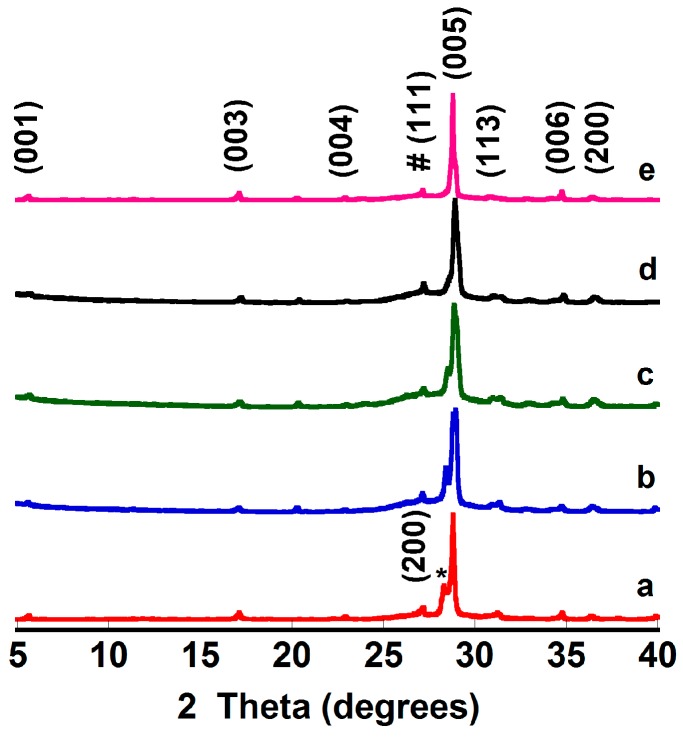
Powder X-ray diffraction (XRD) patterns of thermally-treated [Bi_2_Ba_2_O_4_]_0.5_[CoO_2_] samples as a function of time at 700 °C: (**a**) 0; (**b**) 24; (**c**) 96; (**d**) 384; and (**e**) 1536 h. Crystallographic planes are indicated for the [Bi_2_Ba_2_O_4_]_0.5_[CoO_2_] phase; * Bi-Ba-O solid solution; and # Ge, used as internal reference.

Powder XRD patterns of several representative samples (from 5° to 40° for clarity) are displayed in Figure 1.

From the graph, it is clear that all samples have very similar diffraction patterns, and most of the peaks correspond to the (001) planes of the misfit cobaltite phase, indexed as a monoclinic structure (P2/m space group), and in agreement with previously reported data [15]. The peaks marked by a * correspond to a Bi-Ba-O solid solution with P121/n1 space group [26]. Moreover, the peak appearing at around 27.5° corresponds to the (111) diffraction plane of Ge, used as internal reference (indicated by #). In all cases, it seems that the amount of secondary phases should be very small, and that the thermal treatment decreases their amount, as can be deduced from the lower relative intensity of their (200) peak with time. Moreover, this peak totally disappears for times higher than 384 h. Therefore, all these data clearly indicate that the thermal treatment under simulated working conditions (700 °C under air) leads to an increase of the thermoelectric phase content.

**Figure 2:**
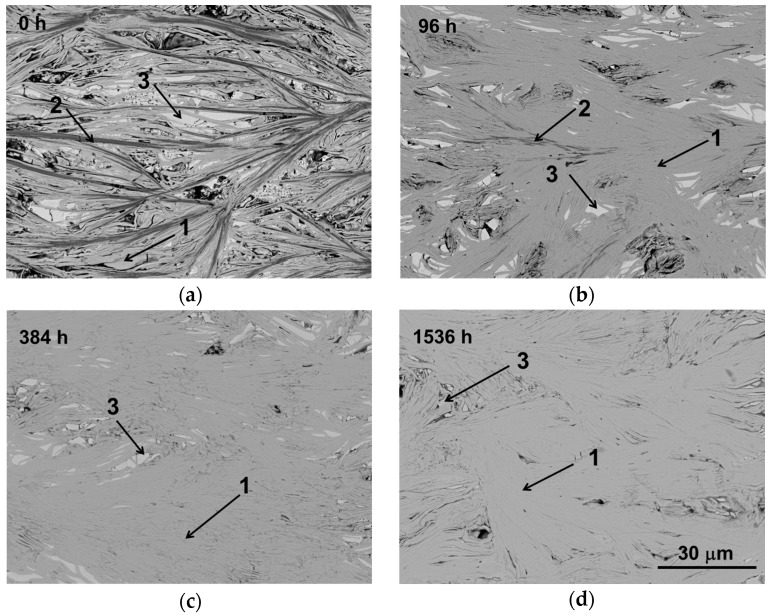
Representative scanning electron microscope (SEM) micrographs of longitudinal polished samples as a function of time at 700 °C: (**a**) 0; (**b**) 96; (**c**) 384; and (**d**) 1536 h. The different contrasts are indicated by numbers: 1, [Bi_2_Ba_2_O_4_]_0.5_[CoO_2_] phase (grey contrast); 2, Ba-Co-O solid solution (dark grey contrast); 3, Bi-Ba-O solid solution (white contrast).

The microstructural evolution of samples as a function of the thermal treatment length is illustrated in Figure 2 (only 0, 96, 384, and 1536 h thermally-treated samples are shown for clarity). At a first sight, a significant change between as-grown (a) and thermally-treated (b, c, and d) samples can be observed. In as-grown samples, three contrasts can be observed, associated through EDS analysis to different phases. Grey contrast (#1) corresponds to the [Bi_2_Ba_2_O_4_]_0.5_[CoO_2_] phase thermoelectric phase, while dark grey and white ones (#2 and #3, respectively) have been identified as Ba-Co-O and Bi-Ba-O solid solutions, respectively.

The amount of secondary phases (contrasts #2 and #3) decreased with the duration of thermal treatment. Moreover, only two contrasts (#1, and #3) can be found for treatments longer than 192 h. The decrease of Bi-Ba-O solid solution and the increase in thermoelectric phase content is in clear agreement with the previously-discussed XRD data. On the other hand, all samples show very low porosity, as they have been directionally grown from the liquid. This process leads to a favourable grain alignment of the TE crystals with their ab-planes along the rod axis (growth direction). The deviation from the growth direction has been measured over different micrographs, and a mean value of ~12° has been obtained. This effect is mainly due to the radial thermal gradients appearing in the solidification front [25]. Moreover, the thermal treatment is not modifying the orientation of the grains.

**Figure 3:**
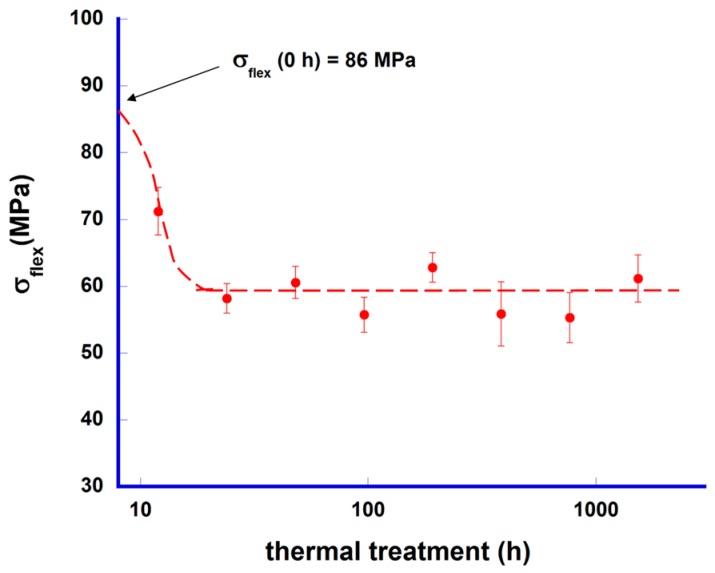
Mechanical performance (three-point bending) of [Bi_2_Ba_2_O_4_]_0.5_[CoO_2_] samples together with their standard error as function of time at 700 °C. Mechanical strength for the as-grown sample (0 h) is marked by an arrow.

To evaluate the mechanical behaviour evolution of samples, flexural strength tests were performed in all samples. The mean values together with their standard errors are presented in Figure 3 as a function of time. In the graph, it can be observed that mechanical strength decreases for very short thermal treatments when compared with the as-grown samples, reaching a nearly constant value for treatments ≥24 h. The broken line is a guide for the eyes, and shows the linear fit of the constant zone. The mechanical evolution can be easily explained by the microstructural modifications produced by the thermal treatment. These modifications are more important for short thermal treatments, when the amount of secondary phases is higher, while longer thermal treatments result in minor microstructural changes with negligible effect on the mechanical response of samples. When compared with other CoO-based TE materials, the maximum flexural strength obtained in this work is lower than that obtained in LFZ-textured Bi-Ca-Co-O or Bi-Sr-Co-O materials (~110 and 130 MPa, respectively) [27,28]. On the other hand, when considering Ca-Co-O materials, flexural strength data ranging from ~20 to 40 MPa for conventional sintered samples [29,30] and from ~200 to 285 MPa for spark plasma sintered ones [30] is found. This fact reflects the brittle nature of these materials, and consequently, the great influence of microstructure on the mechanical behaviour. There is also a large variation in the mechanical data reported in the literature for other interesting TE materials (skutterudittes, half-Heusler alloys, SiGe, PbTe, BiTe, etc.) [31,32,33,34,35] depending on the nature, composition, and preparation methods of the samples.

**Figure 4:**
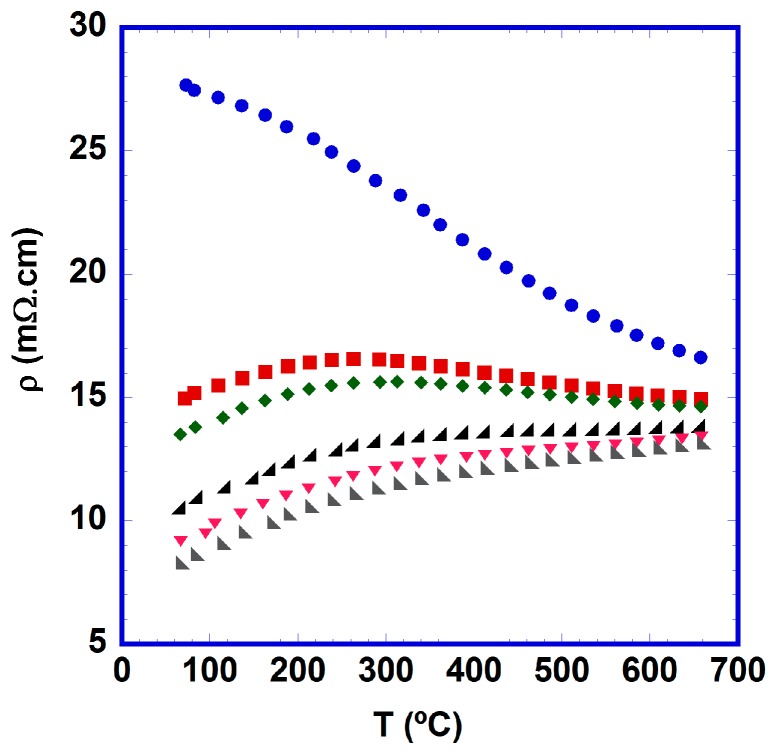
Temperature dependence of the electrical resistivity for [Bi_2_Ba_2_O_4_]_0.5_[CoO_2_] samples as a function of time at 700 °C (●, 0; ■, 12; ◆, 24; ◢, 96; ▼, 384; ◣, 1536 h).

The evolution of electrical resistivity as a function of time at 700 °C is represented in Figure 4.

From these data, it is clear that electrical resistivity is decreased as the duration of thermal treatment is increased. Moreover, these changes are more important in the first few hours of the thermal treatment, in agreement with the rapid decrease of secondary phases in the samples, as has been previously discussed in the mechanical properties section. On the other hand, the electrical characteristics of the samples changed from a semiconducting-like behaviour for the as-grown samples to a more metallic one for the thermally-treated specimens as a result of the increase of the TE phase and the possible oxygenation process produced in the thermal treatment under air. The lowest values were obtained for samples held a 700 °C for 1536 h (~8 and ~13 mΩ·cm at 50 and 650 °C, respectively). The values at room temperature are lower than those previously reported in textured [Bi_2_Ba_2_O_4_]_0.5_[CoO_2_] materials (10–250 mΩ·cm) [16,36,37,38], and relatively close to the values determined in single crystals along the ab plane (4 mΩ·cm) [13,39]. Finally, the high temperature values are clearly lower than the ones found in [Bi_2_Ba_2_O_4_]_0.5_[CoO_2_] sintered materials (33 mΩ·cm) [15,39], and comparable with those obtained in textured samples (between 8 and 50 mΩ·cm) [16,36,38].

**Figure 5:**
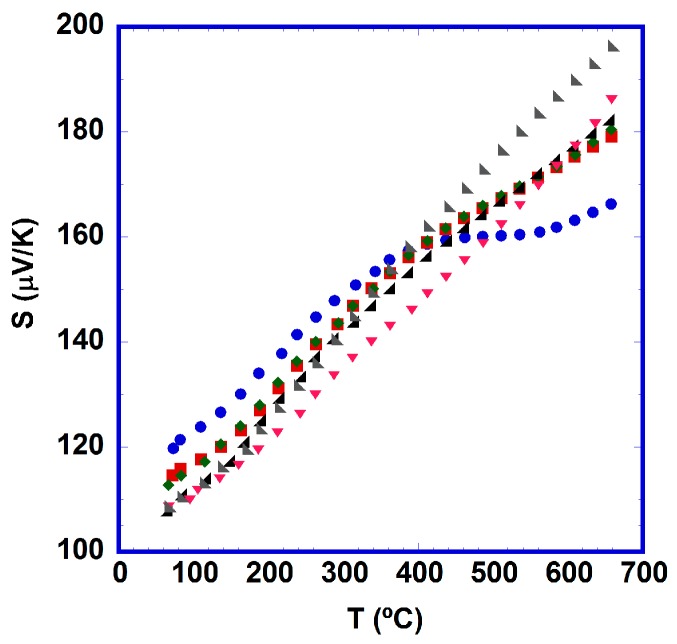
Temperature dependence of the Seebeck coefficient for [Bi_2_Ba_2_O_4_]_0.5_[CoO_2_] samples as a function of time at 700 °C (●, 0; ■, 12; ◆, 24; ◢, 96; ▼, 384; ◣, 1536 h).

Figure 5 displays the evolution of Seebeck coefficient as a function of temperature for all samples. In all cases, *S* is positive in the whole measured temperature range, indicating a conduction mechanism mainly governed by holes.

Moreover, all of the thermally-treated samples display an approximately near-linear increment with the temperature, which can be associated with a metal or degenerated semiconductor typical behavior when the variation of carrier concentration, effective mass, and Fermi level with temperature are negligible. This behavior is in clear agreement with the metallic character of thermally-treated samples found in the electrical resistivity measurements. However, a deviation from this behavior is observed in the as-grown sample, in accordance with the semiconducting-like behavior previously observed in resistivity. Moreover, a slight decrease in Seebeck coefficients at room temperature is obtained as function of time at 700 °C, which clearly agree with a slow increase in oxygen in the structure, and in agreement with Koshibae et al.’s equation [40]. In this way, a Seebeck coefficient value around 120 μV/K was measured for the as-grown sample at room temperature, while all the thermally treated samples exhibit lower ones, reaching the lowest value (~110 μV/K) in the samples aged for the longest times. In any case, all these values are higher than those obtained in sintered materials (80–100 μV/K) [15,37,41,42,43,44,45], and comparable to those measured in single crystals (between 95 and 110 μV/K) [13,39]. On the other hand, they are clearly lower than those measured in well-textured materials thermally treated at higher temperatures for short periods of time (115–145 μV/K) [16,36,37,38]. However, the maximum measured *S* values were obtained at high temperature (650 °C). These values—approximately between 180 and 195 μV/K—are much higher than those reported in sintered (105–120 μV/K) [15,41,45], and slightly higher than those measured in textured (145–185 μV/K) [16,36,38] materials. Consequently, the values obtained in this work are between the highest ever reported in this compound.

**Figure 6:**
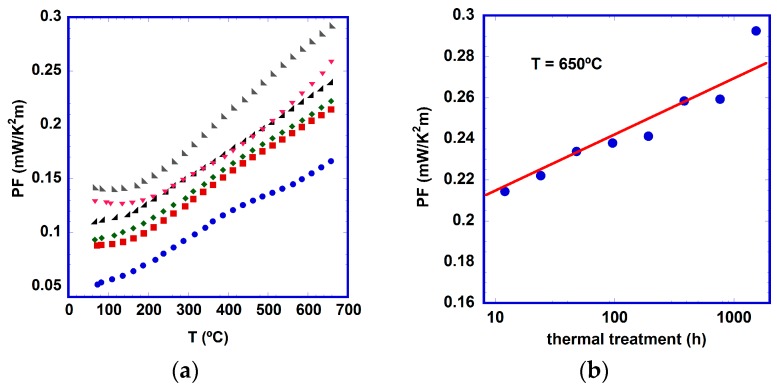
Thermoelectric performance: (**a**) temperature dependence of power factor (*PF*) for [Bi_2_Ba_2_O_4_]_0.5_[CoO_2_] samples as function of time at 700 °C (●, 0; ■, 12; ◆, 24; ◢, 96; ▼, 384; ◣, 1536 h.); and (**b**) *PF* for [Bi_2_Ba_2_O_4_]_0.5_[CoO_2_] samples at 650 °C.

From the electrical resistivity and Seebeck coefficient data, *PF* evolution as a function of temperature was calculated and is displayed in Figure 6a. As can be clearly seen, an increment in the measured TE performance is obtained with longer thermal treatment. These changes are more pronounced in the first few hours of the thermal treatment, in agreement with previous discussions. To better clarify the *PF* evolution as a function of the thermal treatment duration, *PF* values at 650 °C are displayed in Figure 6b, where a slight but continuous improvement of the TE characteristics of samples is observed. A logarithmic fit of these data (indicated by a straight line in the logarithmic scale) has been performed, clearly confirming the former assertion. The maximum *PF* values obtained in this work at 650 °C (~0.29 mW/K^2^m) are close to the best reported ones in LFZ textured materials—0.40 mW/K^2^m when they are grown at very low rates (5 mm/h) [16] or 0.45 mW/K^2^m for Pb-doped ones [38]. When comparing these values with the best ones obtained in sintered materials (0.013–0.040 mW/K^2^m) [15,41,45], the good TE properties of these aged samples at 700 °C are clearly confirmed.

## 4. Conclusions

[Bi_2_Ba_2_O_4_]_0.5_[CoO_2_] rods were successfully prepared by the laser floating zone technique. XRD and SEM characterization showed that the as-grown materials are mainly composed of the thermoelectric [Bi_2_Ba_2_O_4_]_0.5_[CoO_2_] phase, accompanied of two secondary phases identified as Bi-Ba-O and Ba-Co-O solid solutions. When the as-grown samples are aged at 700 °C under air, a fast evolution in the microstructure is observed. After ~384 h, the rods are nearly composed of a single thermoelectric phase. This fact is also reflected in the mechanical and electrical properties, where a rapid change is detected in the first steps of thermal treatment. In the case of the mechanical properties, the flexural strength of these samples reaches a nearly constant value after 24 h, without any further detriment for longer periods of time. TE properties—evaluated through the power factor—show a slight but continuous improvement. This amelioration has been related with the decrease of electrical resistivity of the samples, as the Seebeck coefficient values are practically insensitive to the duration of thermal treatment. Therefore, it has been demonstrated that [Bi_2_Ba_2_O_4_]_0.5_[CoO_2_] rods grown by the LFZ process show a long-term high-temperature stability under air, and they can consequently be regarded as very attractive candidates for practical energy generation applications at high temperatures under air.
